# *TasA-tasB*, a new putative toxin-antitoxin (TA) system from *Bacillus thuringiensis *pGI1 plasmid is a widely distributed composite *mazE-doc *TA system

**DOI:** 10.1186/1471-2164-7-259

**Published:** 2006-10-13

**Authors:** Sarah Fico, Jacques Mahillon

**Affiliations:** 1 Laboratoire de Microbiologie Alimentaire et Environnementale, Université catholique de Louvain, Croix du Sud, 2/12, B-1348 Louvain-la-Neuve, Belgium

## Abstract

**Background:**

Post-segregational killing systems are present in a large variety of microorganisms. When found on plasmids, they are described as addiction systems that act to maintain the plasmid during the partitioning of the cell. The plasmid to be maintained through the generations harbours a group of two genes, one coding for a stable toxin and the other coding for an unstable antitoxin that inhibits the effects of the toxin. If, during cell division, the plasmid is lost, the toxin and antitoxin proteins present in the cytosol cease to be newly expressed. The level of unstable antitoxin protein then rapidly decreases, leaving the toxin free to act on the cellular target, leading to cell death. Consequently, only cells harbouring the plasmid can survive.

**Results:**

The pGI1 plasmid of *Bacillus thuringiensis *H1.1 harbours a group of two genes, one showing similarities with the Doc toxin of the *phd-doc *toxin-antitoxin system, potentially coding for a toxin-antitoxin system. Attempts were made to clone this putative system in the Gram-negative host *Escherichia coli*. The putative antitoxin *tasA *was easily cloned in *E. coli*. However, although several combinations of DNA fragment were used in the cloning strategy, only clones containing a mutation in the toxin gene could be recovered, suggesting a toxic activity of TasB. An exhaustive search was carried out in order to index genes homologous to those of the putative *tasA-tasB *system among microorganisms. This study revealed the presence of this system in great number and in a large variety of microorganisms, either as *tasA-tasB *homologues or in association with toxins (or antitoxins) from other TA systems.

**Conclusion:**

In this work, we showed that the pGI1 plasmid of *B. thuringiensis *H1.1 harbours genes resembling a toxin-antitoxin system, named *tasA-tasB *for *thuringiensis *addiction system. This system appeared to be functional but unregulated in *E. coli*. Bioinformatics studies showed that the *tasAB *system is present on plasmids or chromosomes of a large variety of microorganisms. Moreover, the association between TasA antitoxin with toxins other than TasB (and *vice versa*) revealed the composite and modular nature of bacterial TA systems.

## Background

Plasmids are extrachromosomal elements that carry mostly non-essential genes. However, they often confer advantages to their host, because of determinants such as antibiotic-resistance or virulence genes. When a mother-cell divides, plasmids have to be partitioned into the daughter-cells to keep for the progeny to retain this advantage. For high copy number plasmids, this process occurs by random distribution. However, when a plasmid is present in the cell at a low copy number, the cell has to develop active systems in order to avoid the production of plasmid-free progeny by loss of plasmid during partitioning. One strategy is to actively distribute the plasmid by a mechanism that relies on the presence of centromere-like functions [1,2].

Another strategy is the mechanism called post-segregational killing system or toxin-antitoxin (TA) system. The plasmid to be maintained harbours a group of two genes, one coding for a stable toxin, and the other one expressing the antitoxin counterpart. This antitoxin is an unstable protein that is continuously degraded by a protease. When the plasmid is present in the cell, both toxin and antitoxin are expressed and the antitoxin acts on the toxin to prevent its toxic effects. If, during segregation, the plasmid is lost, toxin and antitoxin are no longer produced and the level of antitoxin rapidly decreases in the cell, leaving the toxin free to act on its target and leading to the death of the cell. Only cells harbouring the plasmid do survive.

Such systems are known to be present in a wide range of prokaryotes [3]. Although the targets of the toxins may differ and sequence homologies may be quite low, these systems tend to be very similar in structure and regulation [4]. One of these systems, *phd-doc*, has been described in bacteriophage P1 as an addiction operon [5]. This temperate bacteriophage is stably maintained as a plasmid prophage in the Gram-negative bacterium *Escherichia coli*. The bacteriophage harbours a group of two genes, one coding for a toxin, *doc *(death on curing), and an upstream gene coding for its antitoxin, *phd *(prevent host death) [5]. In host cells possessing the P1 genome, stable Doc toxin and unstable Phd antitoxin are continuously expressed [6]. Phd antitoxin interacts with the Doc toxin, preventing it from carrying out its molecular action on the target, which is unknown. It has been suggested that a trimeric complex P_2_D is formed, sterically or structurally altering the Doc toxin activity on the target by buffering free Doc molecules within the cell [7]. Moreover, Phd is also known to repress both its own transcription and that of the Doc molecule by binding to an operator DNA site that overlaps the addiction promoter [8]. It has also been suggested that the Phd antitoxin might exert its action by activating another protein that neutralizes the Doc toxin [7]. The Phd antitoxin is continuously degraded in the host cell by the ClpXP protease system [6], composed of the ClpP protease and the regulatory ClpX ATPase subunits [9-11]. *Phd-doc *is thus an active system that ensures stable inheritance of the P1 genome in the host cell population by killing any segregants that are free of the P1 genome.

The actual function of TA systems had to be re-examined, however, when chromosomal TA systems were discovered. A first hypothesis proposes that chromosomal TA systems contribute to programmed cell death (PCD) that occurs in response to various stress signals [12-15]. Indeed, experiments carried out on the *mazEF *system have shown that high levels of ppGpp, the signal molecule of nutritional stress, repress the *mazEF *promoter. This repression leads to the drop of MazE antitoxin levels and subsequently to the death of the cell [16]. By the same mechanism, antibiotics inhibiting transcription, such as rifampicin, or translation, such as chloramphenicol and spectinomycin, have been shown to induce PCD *via *the activation of the *mazEF *system [17]. A second hypothesis suggests that, rather than activating programmed cell death, chromosomal TA systems may induce a reversible bacteriostatic state to protect the cell in response to stressful conditions [3]. Indeed, it was shown that the induction of the antitoxin MazE after exposition to overproduction of the toxin MazF restored the viability of the cell, suggesting that the toxin MazF is bacteriostatic rather than bacteriocide.

*Bacillus thuringiensis *H1.1 is a member of the *Bacillus cereus *group of Gram-positive bacteria. This bacterium occurs naturally in soil and on plants and is considered to be harmless to human. During sporulation, it produces δ-endotoxins that are toxic to insects. *B. thuringiensis *strain H1.1 contains at least four large plasmids (> 30 kb) and three small plasmids: pGI1, pGI2, pGI3 [18]. The complete sequences of pGI2, pGI3 and pGI1 plasmids have been determined [18-20]. Based on the analysis of the 8254 bp pGI1 plasmid, five ORFs larger than 100 aa in size have been identified [18]: a *rep *gene encoding the Rep protein, which is responsible for the autonomous replication of the plasmid, a *mob *gene that enables mobilization of the plasmid, and ORF5, which is a putative transcriptional regulator. Another ORF, downstream of the *mob *gene, encodes a 133-residue protein and shows a high degree of similarity to the Doc toxin of the P1 bacteriophage. The last ORF (95 aa) located upstream of the putative toxin gene could potentially code for the antitoxin counterpart.

## Results and discussion

### TasB of pGI1 from *B. thuringiensis *H1.1 is toxic in *E. coli*

**Figure 1:**
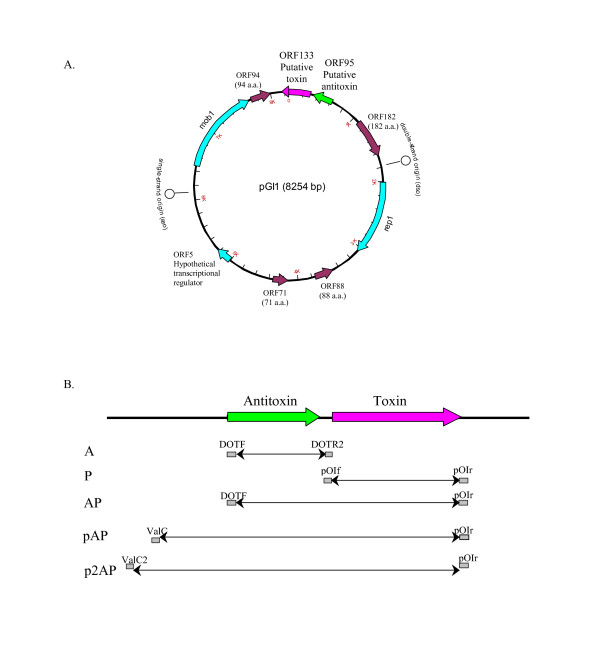
**Organization of toxin and antitoxin genes frompGI1 of *B. thuringiensis *H1.1**. A) pGI1 contains a mobilization gene (*mob1*), a replication gene (*rep1*), a hypothetical transcriptional regulator (ORF5), three small cryptic ORFs (URF94, URF71, URF88) and the putative toxin-antitoxin system *tasB *and *tasA*. TasB displays similarities with the Doc toxin of bacteriophage P1. The upstream ORF, *tasA*, could code for the antitoxin counterpart. Putative double- and single-strand origins are also indicated. B) To assess the function of *tasA *and *tasB*, different parts of this putative toxin-antitoxin system have been amplified for cloning purposes. This figure is a scaled representation of the primers and amplimers used in cloning experiments (See Table 2 for details). Amplimer names are shown in the left column.

pGI1, the smallest plasmid of *B. thuringiensis *H1.1, harbours a pair of ORFs that are predicted to encode 95 and 133 residue proteins, respectively (Fig. 1). The second of these putative proteins displayed sequence similarities (28% identity; E-value = 2e-19 in the Conserved Domain Database) with the Doc toxin of the toxin-antitoxin system *phd-doc *from phage P1. The upstream gene could therefore code for the corresponding antitoxin. This gene pair was tentatively named *tasAB *for *thuringiensis *addiction system.

*TasB*, the putative toxin component of this TA system (fragment P, Table 2) was cloned into the positive-selection vector pCR4-TOPO (Kan^R^, Amp^R^). After electroporation into *E. coli *TOP10, only a few colonies were able to grow on LB + Kan medium. Sequencing these recombinants showed that they all harboured a mutation in the toxin gene (see details below).

Since it was found to be impossible to clone the *tasB *toxin gene alone in *E. coli*, a PCR fragment (AP) spanning the full sequence of both genes from the start codon of the antitoxin *tasA *to the stop codon of the toxin *tasB *was inserted into pCR4-TOPO and transformed into *E. coli*. However, as for *tasB *alone (P fragment), all the recombinant clones contained mutations in the *tasB *toxin gene.

An alternative was to clone the *tasAB *cluster with its own promoter region into *E. coli*. Two constructions were tested: pAP, containing a region of 232 bp upstream of the start codon of antitoxin *tasA*, and p2AP, containing an upstream region of 311 bp. Once again, only recombinants possessing a mutation in the toxin gene *tasB *could be recovered on selective plates.

In order to avoid the killing effect of the TasB toxin, an *E. coli *strain expressing the TasA antitoxin was constructed as follows: the pGI1 *tasA *antitoxin gene was inserted into the positive-selection vector pCR4-TOPO and electroporated in *E. coli *TOP10, leading to the construct pGIF02 (see Material and Methods). The *tasA *antitoxin gene was then inserted into the pCYB10 vector downstream of the IPTG-inducible promoter p_tac _and electroporated into *E. coli *TOP10. Sequencing confirmed the absence of any mutation in the antitoxin gene. The newly constructed vector was named pGIF03. The A, P, AP, pAP and p2AP fragments inserted into vector pCR4-TOPO (see Fig. 1 and Table 2) were then electroporated into the *E. coli *TG1/pGIF03 strain, in the presence of 1 mM of IPTG to induce expression of the antitoxin. Once more, no recombinants harbouring the wild-type *tasB *toxin gene were obtained from the P-, AP-, pAP- and p2AP-containing constructions.

As indicated above, all the recombinant P, AP, pAP and p2AP fragments in *E. coli *harboured mutations in the *tasB *toxin gene. In many cases, the mutation was an early stop codon (Fig. 3). The longest toxin protein obtained in *E. coli *lacked the last 21 amino acids. In other cases, a point mutation leading to the modification of only one amino acid appeared to be sufficient to inactivate the TasB protein. These point mutations were found in all part of the protein but it is interesting to note that four of them (D28, L72, A79 and V80) were found in particularly well conserved regions of the protein TasB. Finally, one case of an IS*1 *insertion in the *tasB *gene was also observed (not shown). All these mutations are reported in the pile-up showing *tasB *and its homologues found in other bacterial genomes (see below) (Fig. 3).

These cloning experiments strongly indicated a toxic effect of TasB when cloned into *E. coli*. This supports the proposal that the pGI1 plasmid of *B. thuringiensis *H1.1 encodes a new putative toxin-antitoxin system. However, this system appeared to be functional but unregulated in *E. coli*. Indeed, the addition of a plasmid containing the *tasA *gene failed to inhibit the lethal activity of TasB in *E. coli*. This may be explained by an inappropriate expression of the antitoxin in the Gram-negative background. TA systems are regulated at transcriptional level by the antitoxin and/or the antitoxin-toxin complex, and this antitoxin is continuously degraded by a specific protease present in the cytosol of the bacterium. It has been shown that the toxin/antitoxin stoichiometry influences the binding of the complex to the promoter-operator region [21]. When cloned into *E. coli*, the *tasA-tasB *system might be misregulated by a change in the TasA/TasB ratio, as a consequence of its dependence on a different system of proteases than in the host *B. thuringiensis*. Experiments are currently underway to determine the TasA level in this bacterium.

### Homologues of the *tasA-tasB *genes of pGI1 reveal the existence of combinatory TA systems in a large variety of microorganisms

#### Homologues of *tasA-tasB *from pGI1 are found in a large variety of microorganisms

The amino acid sequence of the TasB toxin was compared to bacterial protein databases. The genomic location of each toxin homologue was analyzed individually in order to identify any immediately upstream ORF as putative antitoxin. Because antitoxins are often very small proteins, they were not always annotated in the databank. In most cases, a small ORF was indeed present directly upstream of the TasB homologue (Fig. 4).

In 55 cases, the ORF found upstream of the TasB homologue displayed significant similarity to the TasA antitoxin of pGI1. These loci were found in a large variety of microorganism including 14 firmicutes, 26 proteobacteria, 6 chlorobi, 4 cyanobacteria, 2 chlamydiae, 1 acidobacterium, 1 bacteroidete and even 1 planctomycete (see Additional file 2). In this group, all putative antitoxin proteins had about the same size as TasA. Almost all members in this group displayed a gene organization found in many TA systems in which the stop codon of the antitoxin overlaps the start codon of the toxin. This was however not the case for those found in *Bacillus clausii*, *Geobacillus kaustophilus*, *Lactobacillus acidophilus*, *Lactobacillus gasseri*, *Nitrosomonas europaea*, *Rhodospirillum rubrum*, *Rhodopseudomonas palustris *BisB5 and HaA2, *Salinibacter rubber, Xhanthomonas oryzae *KACC10331 and MAFF 311018, *Xhanthomonas campestris vesicatoria *and *Gloeobacter violaceus *(see Additional file 2). In this group, all the TasB homologues were found on the chromosome of the bacteria, except for *Glucunobacter oxydans *621H in which the corresponding locus is found on the plasmid pGOX2 (see Additional file 2).

**Figure 2:**
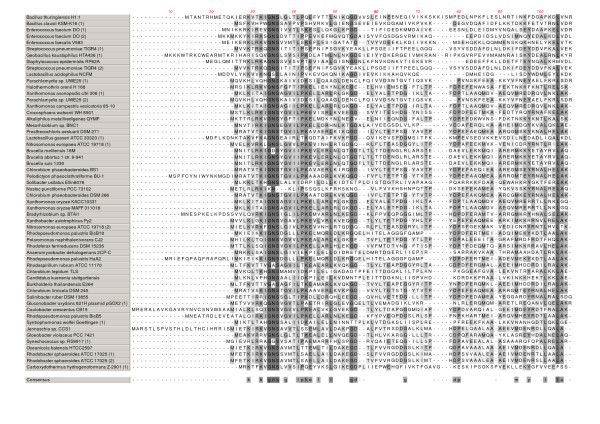
**Putative antitoxins members of the TasAB family**. Multiple alignments of the putative antitoxins related to the TasA antitoxin of pGI1. Only TasA-like proteins with obvious downstream TasB-like partners (see Fig. 4) were included in this comparison. The most conserved amino acids (>50%) are in grey. The names of the bacterial strains where these loci were found are indicated in the left column. The consensus sequence is displayed in the last line.

**Figure 3:**
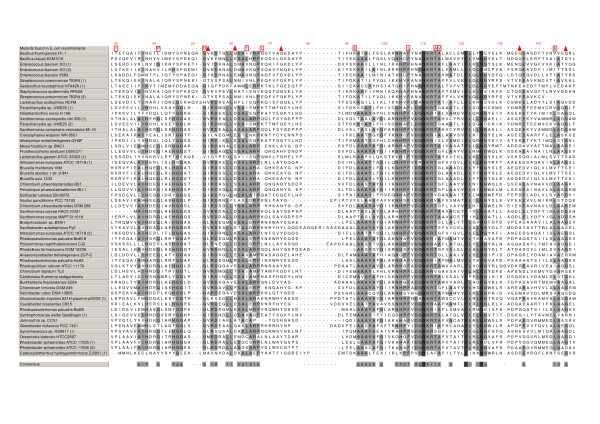
**Putative toxins members of the TasAB family members**. Multiple alignments of the putative toxins related to the TasB toxin of pGI1 from *B. thuringiensis *H1.1. Only TasB-like proteins with obvious upstream TasA-like partners (see Fig. 4) were included in this comparison. The left column shows the bacterial host of the protein. Fully conserved amino acids are in dark grey while the other most conserved residues (>50%) are shown in light grey. Variations observed in the TasB mutants recovered from cloning in *E. coli *are displayed at the top line of the alignment. Many are point mutations (square) and other are early stops (triangle). The consensus sequence is displayed in the last line of the pile-up.

The 55 TasB homologues found in this group were aligned (Fig. 3). The pile-up showed a particularly well conserved region, with the motif H(x)_5_NKR(x)_8_F(x)_3_N. The 55 TasA homologues found upstream of the TasB homologues were also aligned (Fig. 2). It was interesting to observe that the end of the protein is particularly well conserved in all species, with a well conserved C-terminal domain. The GNS motif on the N-terminal part of the protein was also very well conserved among the organisms of this group.

**Figure 4:**
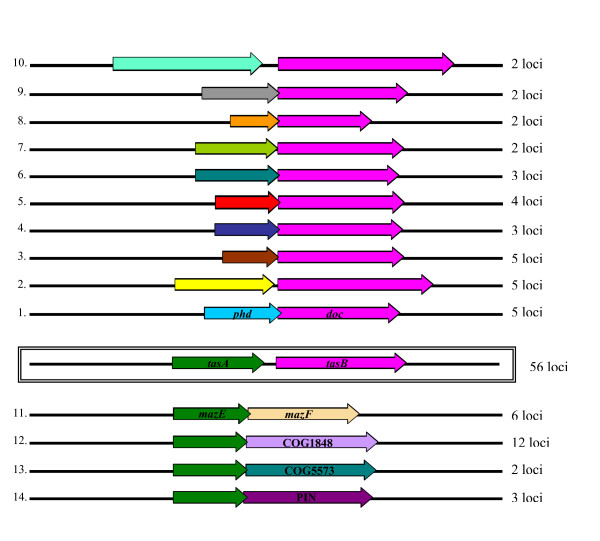
**Groups of loci whose corresponding proteins show similarities with TasA and/or TasB**. Schematic representation of the genomic locations of TasB homologues and their associated upstream ORF, and genomic locations of TasA homologues and their associated downstream ORF. Associated upstream- and downstream genes were grouped according their similarities when blasted against the Conserved Domain Database (CDD). The number of the group (see text for details) is indicated in the left column. The number of loci composing each group is indicated in the right column. Known domains are indicated inside the arrow of the corresponding group.

These 55 loci displaying similarities with both TasA and TasB were grouped into a family named the TasAB family (Fig. 4 and Additional file 2). To our knowledge, the *tasAB *system is the first described in this novel family.

#### TasB homologues can be associated with putative antitoxins unrelated to TasA

In addition to the loci described above, the bioinformatic analysis provided 62 more TasB homologues (E value < 1) that were associated with upstream genes unrelated to the putative antitoxin TasA. These loci were also found in a large variety of microorganisms including 35 proteobacteria, 10 actinobacteria, 8 firmicutes, 4 archaea, 2 cyanobacterium, 1 chloroflexi, 1 chlamydiae and 1 phage (P1). All are chromosomal loci, except for one that was found in the pCC7120epsilon plasmid of *Nostoc sp*. PCC 7120 and one found on the pKLH205 plasmid of *Acinetobacter sp*. ED-4525. The upstream ORFs were compared to the Conserved Domain Database (CDD) [22] and classified according to their similarities with other published sequences or with other upstream genes found in this analysis (see Additional file 3). By this classification, 10 groups of composite TA loci were obtained (Fig. 4).

In the first group (group 1), which was composed of 5 loci, one of the TasB homologues was a confirmed toxin from the known *phd-doc *TA locus of enterobacterial phage P1 [5]. All the systems included in this group harboured an upstream ORF similar (identity > 39%) to the Phd antitoxin of the *phd-doc *system. This group was thus composed of loci displaying similarities to both the TasB/Doc toxins and the Phd antitoxin. The 5 members of this group were all similar in size and organization (translation coupling). The second group (group 2) was composed of five TA loci (identity > 44%) that were all found in γ-proteobacteria. All antitoxins of this group were very similar in size except for the one found in *Ps. aeruginosa*, which was smaller than the others. It is also interesting to note that in the putative toxin gene of the *Ps. aeruginosa *TA locus, two frameshifts give rise to a hybrid protein. Group 3 of composite TA systems included small antitoxins (identity > 66%). However, in two cases, *Vibrio cholerae *O1 eltor N16961 and in *V. cholerae *V52, the putative antitoxin seemed to be fused at its amino-terminal part with a putative acetyl-transferase (not shown). In the next 6 groups (groups 4 to 9), all upstream proteins were similar to each other within a group (identity > 38%), but shared no similarities with other known proteins. The last group (group 10) was composed of 2 members (100% identical) from the same organism *delta proteobacterium *MLMS-1. They were larger than *tasA *and contained a domain similar to that of ParB, which has been proposed to be a nuclease involved in plasmid stability [23].

In addition to these 10 groups, a number of orphan upstream genes were found (see Additional file 2). These genes were predicted to code for proteins displaying no similarity to TasA or to other known genes. It is possible that new groups of antitoxins could be discovered.

Furthermore, nine TasB homologues, all originating from the proteobacteria phylum, were found to be significantly larger than the others (see Additional file 2). While the carboxy-termini of the corresponding proteins were similar to the pGI1 TasB toxin, their amino-termini displayed similarity with the COG3943 domain. This domain is defined in the Conserved Domain Database as related to a virulence protein because of its similarities to RhuM. This protein, located in the SPI-3 pathogenicity island of *S. enterica *[24], is predicted to be a virulence protein because mutants carrying a knocked out copy exhibit diminished ability to invade epithelial cell and/or to induce polymorphonuclear leukocyte migration in a tissue culture model of mammalian enteropathogenesis [25]. In this group, no upstream antitoxin seemed to be associated with these putative toxins.

Finally, 10 TasB homologues were defined as "solitary toxin" [26], since they had the same size as TasB but were apparently not associated with an antitoxin partner (not shown). These solitary toxins were found in a broad range of microorganisms including 4 proteobacteria, 2 actinobacteria, 2 archaea, 1 fusobacterium and even 1 fungus. The latter was found in *Aspergillus fumigatus*. This gene has been annotated as a putative member of the Doc family ([GenBank:EAL85381], Nierman *et al*., unpublished), but the exact function of the protein has not been demonstrated. If this homologue were proved to be functional, this would be the first TA locus identified in a fungal species.

Similar cases of solitary genes have already been reported in the study of homologues of the YdcE toxin (MazF family) from the *ycDE *operon of *Bacillus subtilis *[27], in a study describing a number of TA loci from the vapBC family (vapC is a toxin containing a PIN domain) [28] and in an exhaustive study of TA homologues [26]. This suggests that if those solitary toxins were found to be functional, other mechanisms of regulations specific to each species would have to exist.

#### TasA homologues can be associated with either TasB homologues or with other toxin families

As in the case of the TasB toxin, the TasA antitoxin of pGI1 was compared to potential downstream ORFs for their similarities and putative functions. Homologues of the TasA antitoxin found in this study were separated into three classes: those with a TasB-like downstream gene as described above (Fig. 2 and Fig. 4), the second including TasA homologues with a toxin-like downstream gene (Fig. 4), and TasA homologues not associated with a putative toxin.

Homologues of TasA associated with proteins unrelated to TasB were found in 15 archaea, 6 proteobacteria, 4 firmicutes and 1 spirochaete (see Additional file 2). Downstream proteins of TasA homologues were grouped according to their similarities (see Additional file 3); four groups of composite TA loci were obtained (see Additional file 2). The first group (group 11) was composed of 6 TA loci (identity > 27%) very similar in size, originating from phylogenetically distant species, and all sharing similarities with the *mazF *toxin of the confirmed TA locus *mazEF*. This high similarity between TasB and the Doc toxin (28% identity ; E-value = 2e-19 in the CDD), combined with the similarity between TasA and the MazE antitoxin (23% ; E-value = 2e-3 in the CDD), indicated that the *tasA-tasB *toxin-antitoxin system from pGI1 is thus a composite *mazE-doc*-like system. In the second group (group 12), 12 TA loci were found (identity > 27%). The putative toxins downstream of the TasA-like genes were all similar in size, except for the one originating from *Pyrococcus horikoshii*, smaller than the others and than the TasB toxin from pGI1. All the putative toxins found in this group belonged to the conserved domain COG1848. Proteins of this group are predicted nucleic acid-binding proteins, containing a PIN domain. The PIN (PilT N-terminus) domain was first annotated on the basis of sequence similarity to the N-terminal domain of the pilT protein from *Myxococcus xanthus *[29]. Proteins containing such a domain can be found in the genome of a large variety of prokaryotes and eukaryotes. By analogy with eukaryotic PIN proteins, which are ribonucleases [30], prokaryotic PIN proteins have been predicted to be toxic components of chromosomally encoded TA operons [31]. The *vapBC *locus of *Leptospirra interrogans *has been described as a toxin-antitoxin system in which the VapC toxin contains a PIN domain [32].

Both loci of the next group (group 13) harboured a conserved domain COG5573. This group is related to the COG1848 group and also contains a PIN domain. The last group (group 14) contained 3 loci of which the downstream proteins belonged to the pfam01850 group, also harbouring a PIN domain. These 3 groups were thus considered to be functionally related as they all contained a PIN domain. In addition to these groups, three orphan cases of composite TA loci were found in which the associated toxin showed no similarity with other toxins (see Additional file 2).

In addition to those found above, a number of TasA homologues were found for which no gene could be identified immediately downstream of their genomic location (not shown). These genes were often longer than the antitoxin of pGI1. Only the N-part of the protein displayed a high degree of homology with the antitoxin. These homologues have been annotated as transcriptional regulator of the AbrB- and SpoVT-family. Comparison with the CDD (Conserved Domain Database, NCBI) showed that they all harboured a SpoVT/AbrB-like domain [22]. The product of the AbrB gene is an ambiactive repressor and activator of the transcription of genes expressed during the transition state between vegetative growth and the onset of stationary phase and sporulation [33]. AbrB is thought to interact directly with the transcription initiation regions of genes under its control [34]. The product of the *B. subtilis *gene *spoVT *is another member of this family and is also a transcriptional regulator [35].

It has been shown that antitoxins contain motifs common to different classes of DNA-binding proteins and can therefore be classified according to their structural homologies: MetJ/Arc superfamily and related ribbon-helix-helix fold proteins, Phd/YefM and AbrB/MazE superfamilies [36]. Multiple alignments of the TasA homologues showed a highly conserved amino-terminal domain (Fig. 2). This was consistent with other studies that have used mutational analyses to show that antitoxins bind to DNA through their N-terminal domain [3].

## Conclusion

In this work, we showed that the pGI1 plasmid of *B. thuringiensis *H1.1 encodes a new toxin-antitoxin system, called *tasA-tasB *for *thuringiensis *addiction system. This system appeared to be functional in *E. coli*, and a single mutation in the *tasB *gene was sufficient to inhibit the lethal activity of the toxin in *E. coli*. However, the addition of a plasmid containing the *tasA *gene failed to restore the viability of the cells, probably due to an inappropriate expression in the Gram-negative background.

Additionally, our study revealed a new family of TA loci, the TasAB family which is presently composed of at least 56 members, found in a large variety of microorganisms. TA systems have been extensively described and their toxins have been classified into superfamilies, according to their structural homologies: the MazF/Kid/CcdB, the RelE/ParE, the Doc and the PIN superfamilies [36]. In the TasAB family, exhaustive protein sequence searches showed that the TasB toxin of the *tasAB *system from pGI1 is similar to Doc of the *phd-doc *system. Interestingly, TasA was not similar to the antitoxin Phd, but presented similarities with the MazE antitoxin from the *mazEF *TA system. The tasAB system and members of its family can thus be viewed as hybrid systems between the *phd-doc *and the *mazEF *systems. Moreover, other associations between toxins and antitoxins from different families can be found, as illustrated in our extensive protein search which showed that TasA and TasB homologues can be found in association with partners from other TA systems.

The present study confirmed that more than multiple TA systems can be found in the same bacterium. In the *Sulfolobus solfataricus *genome for exemple, 22 TA loci, all from the *vapBC *family (vapC is a member of the PIN domain family) have been found [28]. Interestingly, microarray experiments have revealed the implication of these TA loci in the heat shock response, which involves the modulation of their expression under stress conditions [28]. Since TA system could be stress-response elements, it has been suggested that free-living organisms, which grow slowly and are exposed to many environmental changes in comparison to host-associated organism, would benefit from having many TA loci [26].

There seems to be a common organization within the TA systems, featuring two genes, one coding for a toxin and the other coding for a DNA-binding protein that functions as an antitoxin and a transcription factor. Because composite associations of different toxins and antitoxins were found, it has been suggested that TA systems do not descend from a common ancestor but have been assembled from different proteins which can be displaced by functional equivalents, while the operon architecture itself is preserved [36]. Consistently with this idea, ORF of unknown function associated with a TasB homologue could potentially represent members of new antitoxin families.

## Methods

### Bacterial strains, plasmids and growth conditions

Table 1 reports the bacterial strains used in this study, including their origin, reference and main characteristics. The *B. thuringiensis *and *E. coli *strains were grown on Luria-Bertani (LB) broth, at 30°C and 37°C, respectively.

The primary cloning of the toxin and antitoxin genes was performed using the kanamycin-resistance, positive-selection plasmid vector pCR4-TOPO (InVitrogen), and electroporated into *E. coli *TOP10 strain. The antitoxin gene was subsequently cloned in the ampicillin-resistance plasmid pCYB10 and the toxin gene in the chloramphenicol-resistance plasmid pBAD33 (see PCR and cloning strategies). Newly constructed plasmids were electroporated in *E. coli *TG1 strains. The antibiotics were used at the following concentrations: 50 μg/ml kanamycin (Kan), 100 μg/ml ampicillin (Amp), and 15 μg/ml chloramphenicol (Cm).

### DNA preparation

Total DNA from *B. thuringiensis *H1.1 was prepared using standard protocol and stored at -20°C. Plasmid DNA preparations were obtained using the High Pure Plasmid Kit (Roche).

### PCR and cloning strategies

#### Cloning fragments of the toxin-antitoxin system from pGI1 of *B. thuringiensis *H1.1 in pCR4-TOPO

Table 2 shows the oligonucleotide primers (purchased from Sigma-Genosys) used in the PCR method for cloning different gene fragments of the toxin-antitoxin system from pGI1 of *B. thuringiensis *H1.1. One pair of primer amplified the toxin gene only (P), while another pair amplified a region from the start of the antitoxin gene to the stop of the toxin gene (AP). A third pair flanked the toxin and antitoxin genes with an upstream region thought to contain the promoter region (pAP). Finally, the last pair of primers amplified an additional 78 bp region upstream of the antitoxin gene (p_2_AP) (Fig. 1B). One μl of total DNA (50-fold dilution) of *B. thuringiensis *H1.1 was used for the amplification of the gene fragments in a 50 μl mixture containing 1 μl of DyNAzyme I DNA polymerase (FINNZYME), 5 μl of the 10 × standard buffer, 0,5 μl dNTP (20 mM) and 5 μl primers (10 μM). The reaction was performed in a Perkin Elmer GeneAmp PCR System 9600 thermal cycler using the following program: initial denaturation at 96°C for 10 min, 30 cycles of denaturation at 92°C for 1 min, annealing for 1 min at 50°C and extension at 72°C for 1 min 30 sec. An additional extension step for 10 min at 72°C ends the amplification program.

The PCR products were ligated into the plasmid vector pCR4-TOPO using the protocol described in the TOPO TA cloning Kit for sequencing (InVitrogen) and transformed by electroporation into *E. coli *TOP10. Inserts from the Kan^R ^recombinants were sequenced by Genome Express (France) using the M13 universal primers.

#### Cloning of the antitoxin gene in pCYB10

The pCR4-TOPO plasmid containing the A fragment (Fig. 1B), obtained as described above, was sequenced to assess the absence of any mutation, and called pGIF02. Both pGIF02 (Kan^R^, Amp^R^) and pCYB10 plasmids (Amp^R^) were restricted by *Nde*I and *Sal*I enzymes. After extraction on gel using Quantum Prep Freeze'N Squeeze DNA Gel Extraction Spin Column (Biorad), the restricted fragments were ligated using 2 units of T4 DNA ligase (Fermentas, T4 DNA ligase Rapid ligation kit). The newly constructed plasmid, pGIF03 obtained in *E. coli *TG1 was sequenced by Genome Express (France).

### Bioinformatics

#### Search for TasA and TasB homologues

The amino acid sequence of the putative TasB toxin was compared using standard BLASTP . The cut-off *E-*value used in this analysis was 1. The genomic regions carrying potential toxin homologues were analyzed individually using DS Gene 1.5 (Accelrys), in order to find any upstream ORF. The same method was applied to the TasA antitoxin of pGI1 from *B. thuringiensis *H1.1, to find potential downstream partners. All the loci found in this computational study are listed in the Additional file 1.

#### Classification of putative toxins and antitoxin partners

Each immediate upstream ORF of TasB homologue, and downstream ORF of TasA homologue were translated *in silico*, using DS Gene 1.5, and compared to the Conserved Domain Database [22] in order to search for similarity with other known antitoxins/toxins from TA systems or with other known proteins. Identity percentages were calculated within a group by using NCBI/BLAST/align2sequences.

## Authors' contributions

SF carried out the molecular genetic studies and the bioinformatics analyses, participated in the design of the study and drafted the manuscript. JM conceived of the study, and participated in its design and coordination and helped to draft the manuscript. All authors read and approved the final manuscript.

## Supplementary Material

Additional File 2 **Genetic organization of genomic loci whose corresponding proteins show similarities with TasA and/or TasB**. Detailed and scaled representation of genomic loci harbouring a *tasA *and/or a *tasB *homologue. The bacterial hosts of the loci are indicated in the right column, together with the taxonomic groups they belong to. On the left, "P" indicates that the locus is found on a plasmid; others are chromosomal loci. Genes whose corresponding proteins show similarities with both TasA antitoxin and TasB toxin of pGI1 are shown in green and pink, respectively. The upper part of the scheme shows a scaled representation of the genomic locations of TasB homologues (in pink) and their associated upstream ORF. Based on their putative antitoxin similarities, these TA loci could be classified into 10 groups shown with distinct colours. Genes in white are orphan genes whose corresponding proteins share no similarity with other known proteins. In several cases, larger genes encode proteins whose C-terminal ends are unrelated to the TasA antitoxin, but display similarities with a putative virulence protein (grey). The dashed red box in the toxin of *Ps. aeruginosa *refers to a double frameshift (see text for details). The middle part of the picture indexes all members of the TasAB family, where all loci harbour homologies with both TasA and TasB. The lower part of the figure shows a scaled representation of the genetic organization of *tasA *homologues and their associated downstream ORF. These putative TA systems are displayed as 4 groups on the basis of their putative toxins. Genes in white are orphan genes whose corresponding proteins do not share significant similarities with other proteins. Click here for file

Additional File 3 **Alignments of groups of upstream- and downstream proteins of TasB and TasA homologues**. Bioinformatics analyses yielded 10 groups of upstream proteins and 4 groups of downstream proteins (see text for detail). Alignments of each group (1 to 14) are represented in this figure. The names of the strains where these loci were found are indicated in the left column. The consensus sequence is displayed in the last line. Fully conserved amino acids are in dark grey while the other most conserved residues (>50%) are shown in light grey. Click here for file

Additional File 1 **Accession numbers**. Accession number of all proteins (TasB homologues, TasA homologues, and their associated proteins) are listed in this table. Click here for file

## Figures and Tables

**Table 1:** Strains and plasmids. Strains and plasmids used in this study, including their origin, reference and main characteristics

Strains or plasmids	Relevant features	Construction, source or reference
*Strains*		
*B. thuringiensis *H1.1	Isolated from *Ephestia kühniella*, Canada	IEBC (International Entomopathogenic *Bacillus *Center, Pasteur Institute, Paris, France)
*E. coli *TOP10	Cloning host	InVitrogen
*E. coli *TG1	Cloning host	Gibson, 1984
*Plasmids*		
pGI1	Resident plasmid from *B. thuringiensis *H1.1	Mahillon and Seurinck, 1988
pCR4-TOPO	Positive-selection cloning vector, Amp^R^, Kan^R^	InVitrogen
pCYB1	Cloning vector, Amp^R^, IPTG-inducible p_tac _promoter	New England Biolabs Inc.
pCYB10	pCYB1 deleted of intein gene	J. Mahillon and F. Bilocq unpubl. results
pBAD33	Cloning vector, Cm^R^, arabinose-inducible promoter p_BAD_	Guzman *et al*., 1995
pGIF02	PCR fragment containing antitoxin gene of pGI1 cloned into pCR4-TOPO	This study
pGIF03	*Nde*I-*Sal*I pGIF02 fragment containing the antitoxin gene A cloned into pCYB1O	This study
pGIF04Mx	PCR fragment containing the toxin gene P cloned into pCR4-TOPO, 4 clones obtained M1 → M4	This study
pGIF06Mx	PCR fragment containing the antitoxin-toxin locus AP cloned into pCR4-TOPO, 4 clones obtained M1 → M4	This study
pGIF08M1	PCR fragment containing the antitoxin-toxin locus pAP cloned into pCR4-TOPO	This study
pGIF10Mx	PCR fragment containing the antitoxin-toxin locus p2AP cloned into pCR4-TOPO, 15 clones obtained M1 → M15	This study

**Table 2:** Oligonucleotide primers. Oligonucleotide primers used in PCR assays to clone different segments of the toxin-antitoxin system from pGI1.

**Segment**	**Region amplified (Accession number : **AY138809)**^a^**	**Oligonucleotide primers^b^**	**Primer names**	**Length (bp) of amplified region**
A	(304–629)^C^	5'-TTAGG**CATATG**ACAGCAAACACTCGCCACATGG-3'	DOTF	345 bp
		5'-AATAT**GTCGAC**TCATTTCGGTTTTCCTACTTTCCTCC-3'	DOTR2	
P	(8147–8254,1–332)^C^	5'-GGCCC**GGTACC**GTGGAGGAAAGTAGGAAAACCG-3'	pOIf	459 bp
		5'-CCC**GTCGAC**CCCACGTCTTTTTTTAATTTAAAGG-3'	pOIr	
AP	(8147–8254,1–629)^C^	5'-TTAGG**CATATG**ACAGCAAACACTCGCCACATGG-3'	DOTF	754 bp
		5'-CCC**GTCGAC**CCCACGTCTTTTTTTAATTTAAAGG-3'	pOIr	
pAP	(8147–8254,1–861)^C^	5'-AACGGTTAATACGTTCTAACTG-3'	*ValC*	*978 bp*
		5'-CCC**GTCGAC**CCCACGTCTTTTTTTAATTTAAAGG-3'	*pOIr*	
p_2_AP	(8147–8254,1–939)^C^	5'-GGCTCTAGAATCTTCCCTATC-3'	ValC2	*1056 bp*
		5'-CCC**GTCGAC**CCCACGTCTTTTTTTAATTTAAAGG-3'	pOIr	
RTA	(370...630)^C^	5'-AATGACAGCAAACACTCGCC-3'	RTAf	261 bp
		5'-GCACCGTCAAATTTATTGATGG-3'	RTAr	
RTP	(8249–8254,1–228)^C^	5'-GAGGTGTTAAAGATACAGG-3'	RTPf	234 bp
		5'-AATCTGAAGCTTCTTGTTCGG-3'	RTPr	
RTAP	(8249–8254,1–630)^C^	5'-AATGACAGCAAACACTCGCC-3'	RTAf	636 bp
		5'-AATCTGAAGCTTCTTGTTCGG-3'	RTPr	

a: C indicates complementary sequences.

b: Letters in bold refer to restriction sites.
