# Supplementary figures and images for: TasA-tasB, a new putative toxin-antitoxin (TA) system from Bacillus thuringiensis pGI1 plasmid is a widely distributed composite mazE-doc TA system
Source: BMC Genomics. 2006 Oct 13;7:259. doi: 10.1186/1471-2164-7-259 (PMC1626090; doi:10.1186/1471-2164-7-259)

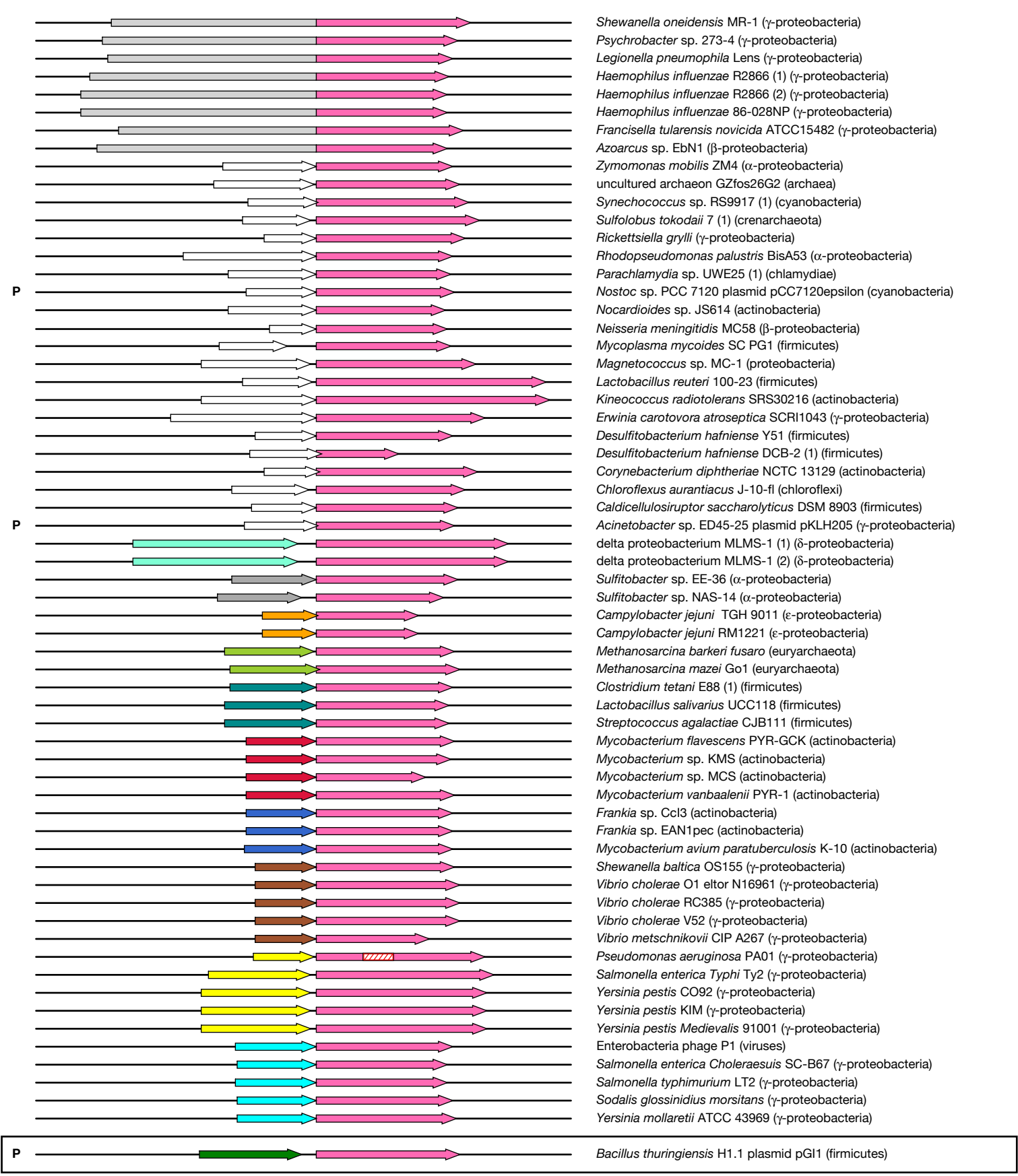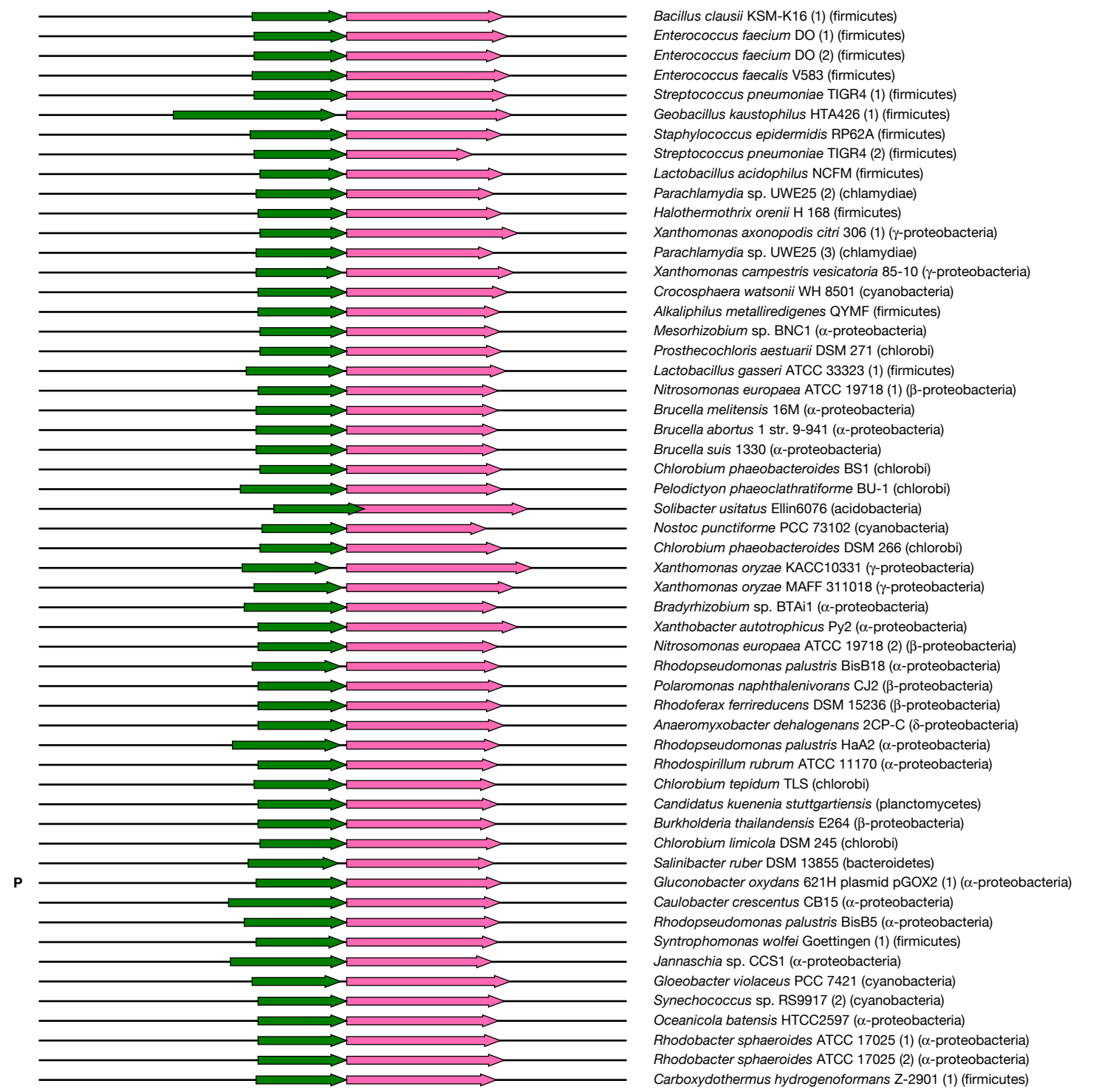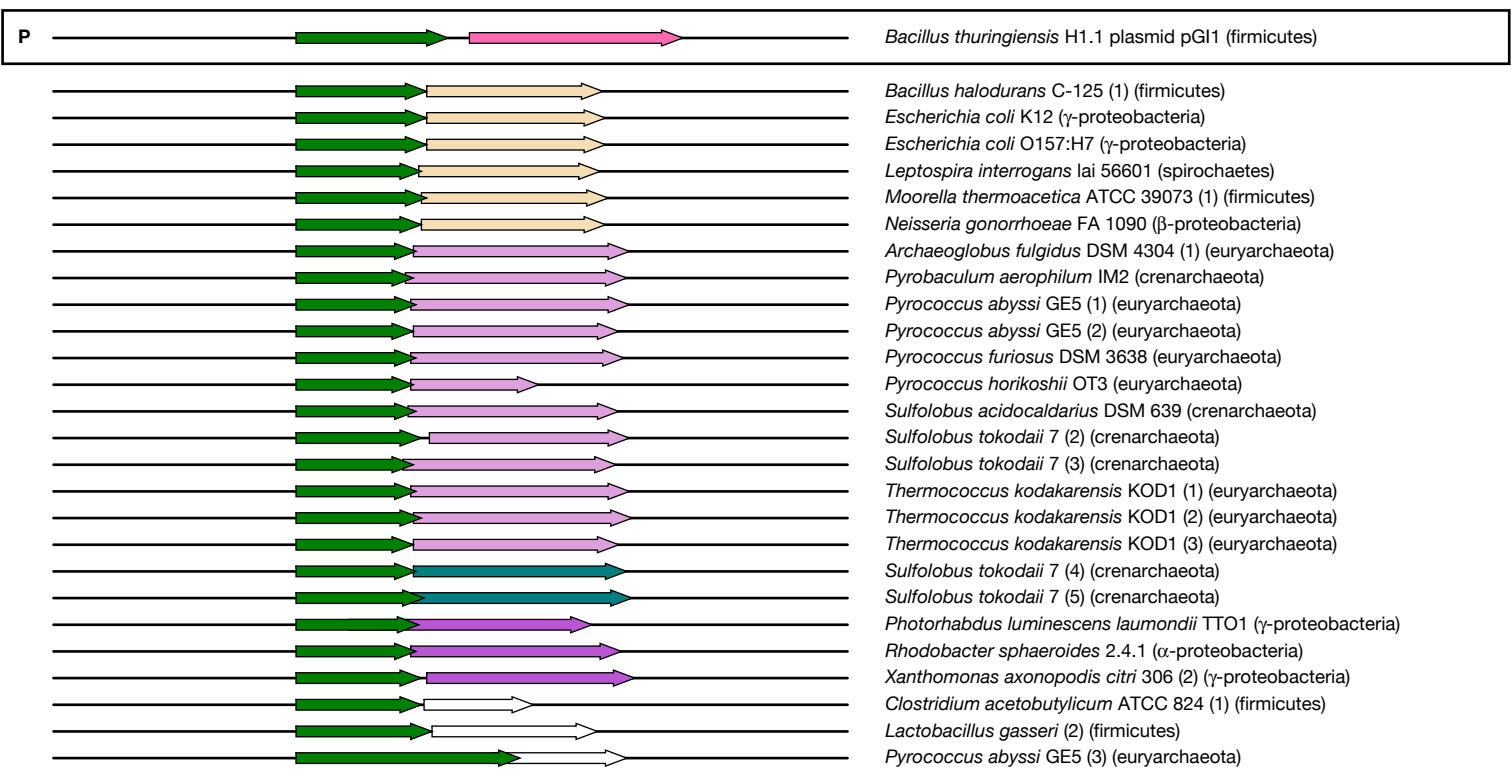

Supplement: Additional File 2 — Genetic organization of genomic loci whose corresponding proteins show similarities with TasA and/or TasB. Detailed and scaled representation of genomic loci harbouring a tasA and/or a tasB homologue. The bacterial hosts of the loci are indicated in the right column, together with the taxonomic groups they belong to. On the left, "P" indicates that the locus is found on a plasmid; others are chromosomal loci. Genes whose corresponding proteins show similarities with both TasA antitoxin and TasB toxin of pGI1 are shown in green and pink, respectively. The upper part of the scheme shows a scaled representation of the genomic locations of TasB homologues (in pink) and their associated upstream ORF. Based on their putative antitoxin similarities, these TA loci could be classified into 10 groups shown with distinct colours. Genes in white are orphan genes whose corresponding proteins share no similarity with other known proteins. In several cases, larger genes encode proteins whose C-terminal ends are unrelated to the TasA antitoxin, but display similarities with a putative virulence protein (grey). The dashed red box in the toxin of Ps. aeruginosa refers to a double frameshift (see text for details). The middle part of the picture indexes all members of the TasAB family, where all loci harbour homologies with both TasA and TasB. The lower part of the figure shows a scaled representation of the genetic organization of tasA homologues and their associated downstream ORF. These putative TA systems are displayed as 4 groups on the basis of their putative toxins. Genes in white are orphan genes whose corresponding proteins do not share significant similarities with other proteins. [file 1471-2164-7-259-S2.pdf]
