# Supplementary material for: TasA-tasB, a new putative toxin-antitoxin (TA) system from Bacillus thuringiensis pGI1 plasmid is a widely distributed composite mazE-doc TA system
Source: BMC Genomics. 2006 Oct 13;7:259. doi: 10.1186/1471-2164-7-259 (PMC1626090; doi:10.1186/1471-2164-7-259)
Supplement: Additional File 3 — Alignments of groups of upstream- and downstream proteins of TasB and TasA homologues. Bioinformatics analyses yielded 10 groups of upstream proteins and 4 groups of downstream proteins (see text for detail). Alignments of each group (1 to 14) are represented in this figure. The names of the strains where these loci were found are indicated in the left column. The consensus sequence is displayed in the last line. Fully conserved amino acids are in dark grey while the other most conserved residues (>50%) are shown in light grey. [file 1471-2164-7-259-S3.pdf]

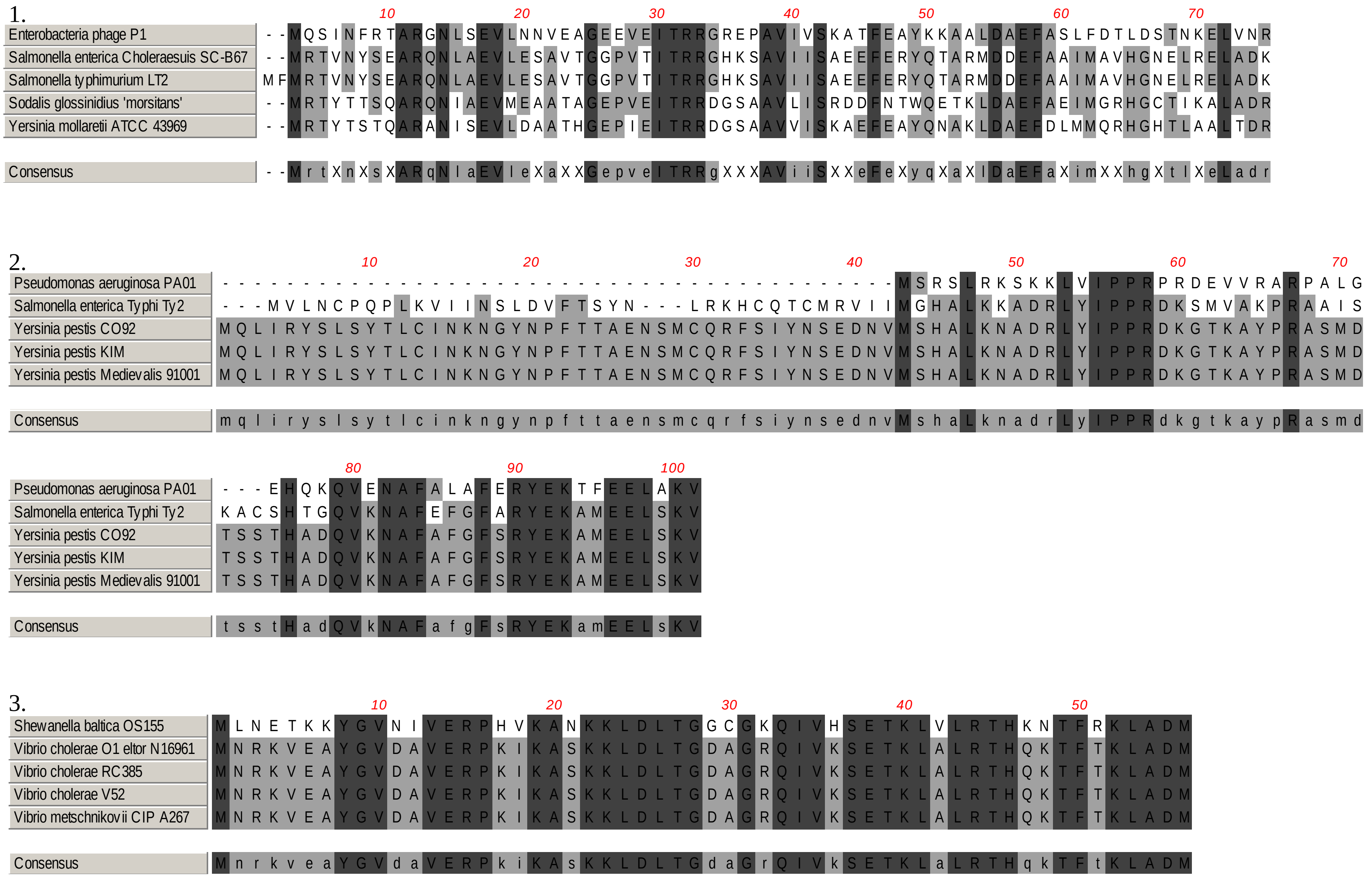
1.
Enterobacteria phage P1 --MQSINFRTARGNLSEVLNNVEAGEEVEITRRGREPAVIVSKATFEAYKKAALDAEFASLFDTLDSTNKELVNR
Salmonella enterica Choleraesuis SC-B67 --MRTVNYSEARQNLAEVLESAVTGGPVTITRRGHKSAVIISAEEFERYQTARMDDEFAAIMAVHGNELRELADK
Salmonella typhimurium LT2 MFMRTVNYSEARQNLAEVLESAVTGGPVTITRRGHKSAVIISAEEFERYQTARMDDEFAAIMAVHGNELRELADK
Sodalis glossinidius 'morsitans' --MRTYTTSQARQNIAEVMEAATAGEPVEITRRDGSAAVLISRDDFNTWQETKLDAEFAEIMGRHGCTIKALADR
Yersinia mollaretii ATCC 43969 --MRTYTSTQARANISEVLDAATHGEPIEITRRDGSAAVVISKAEFEAYQNAKLDAEFDLMMQRHGHTLAALTDR
Consensus --MrtXnXsXARqNlaEVleXaXXGepveITRRgXXXAViiSXXeFeXyqXaXlDaEFaXimXXhgXtlXeLadr
2.
Pseudomonas aeruginosa PA01 --MSRSLRKSKKLVIPPRPRDEVVRARPALG
Salmonella enterica Typhi Ty2 ---MVLNCPQPLKVIINSLDVFTSYN---LRKHCQTCMRVIIMGHALKKADRLYIPPRDKSMVAKPRAAIS
Yersinia pestis CO92 MQLIRYSLSYTLCINKNGYNPFTTAENSMCQRFSIYNSEDNVMSHALKNADRLYIPPRDKGTKAYPRASMD
Yersinia pestis KIM MQLIRYSLSYTLCINKNGYNPFTTAENSMCQRFSIYNSEDNVMSHALKNADRLYIPPRDKGTKAYPRASMD
Yersinia pestis Medievalis 91001 MQLIRYSLSYTLCINKNGYNPFTTAENSMCQRFSIYNSEDNVMSHALKNADRLYIPPRDKGTKAYPRASMD
Consensus mqlirysIsytlcinkngynpfttaensmcqrfsiynsednvMshaLknadrLyIPPRdkgtkaypRasmd
Pseudomonas aeruginosa PA01 ---EHQKQVENAFALAFERYEKTFEELAKV
Salmonella enterica Typhi Ty2 KACSHTGQVKNAFEFGFARYEKAMEELSKV
Yersinia pestis CO92 TSSTHADQVKNAFAFGFSRYEKAMEELSKV
Yersinia pestis KIM TSSTHADQVKNAFAFGFSRYEKAMEELSKV
Yersinia pestis Medievalis 91001 TSSTHADQVKNAFAFGFSRYEKAMEELSKV
Consensus tsstHadQVkNAFafgFsRYEKamEELsKV
3.
Shewanella baltica OS155 MLNETKKYGVNIVERPHVKANKKLDLTGGCGKQIVHSETKLVLRTHKNTFRKLADM
Vibrio cholerae O1 eltor N16961 MNRKVEAYGVDAVERPKIKASKKLDLTGDAGRQIVKSETKLALRTHQKTFTKLADM
Vibrio cholerae RC385 MNRKVEAYGVDAVERPKIKASKKLDLTGDAGRQIVKSETKLALRTHQKTFTKLADM
Vibrio cholerae V52 MNRKVEAYGVDAVERPKIKASKKLDLTGDAGRQIVKSETKLALRTHQKTFTKLADM
Vibrio metschnikovii CIP A267 MNRKVEAYGVDAVERPKIKASKKLDLTGDAGRQIVKSETKLALRTHQKTFTKLADM
Consensus MnrkveaYGVdaVERPkiKAsKKLDLTGdaGrQIVkSETKLaLRTHqkTFtKLADM

4.

	10 20 30 40 50 60
Frankia sp. CcI3	M T L R L T D D E A E A L R L R S E L E Q R S M Q E I A R E A I R E Y I E A H S R A E L L D Q V L D E E L P R Y A E A L R W L G E -
Frankia sp. EAN1pec	M T L R L T D D E T E A L R L R S E L E R R S M Q E I A R Q A I R E Y I E A H S R A E L L D Q V L D E E L P R Y A E A L R R L G E -
Mycobacterium avium paratuberculosis K-10	M T L R P S E Q Q A E A L R R Q A T A E G R S M Q A V A L S A I D E Y I A R R A H K T K V A A A L Q W V V R E E A G V L E R L K D A
Consensus	M T L R l t d d e a E A L R l r s e l E X R S M Q e i A r X A I r E Y I e a h s r a e l l d q v L d e e l p r y A e a L r r L g e -

5.

	10 20 30 40 50 60
Mycobacterium flavescens PYR-GCK	M P A L N I E F T D D E L A T V R A A A S G S G T S M R A F A R Q A I L D R A V D R E G R I R A L G E E I A Q R S A E L N Q R L A
Mycobacterium sp. KMS	M P S L N V D F D D A E M E Q I R A A A K Q D D L S L K K F V H A A V M E R A S M H K R R V A E A A R V V A E R S A E L N R R L A
Mycobacterium sp. MCS	M P S L N V D F D D A E M E Q I R A A A K Q D D L S L K K F V H A A V M E R A S M H K R R V A E A A R V V A E R S A E L N R R L A
Mycobacterium vanbaalenii PYR-1	M P S L N V S F T E E E L A T V R A A A A N D E V S L R T F A H A A I L S A A S E H R R R V A E A A R I V A E R S A E L N R R L A
Consensus	M P s L N v X F X d X E X X X X R A A A X X d X X S l X X F X h a A X X X r A s X h X r R v a e a a r X v A e R S A E L N r R L A

6.

	10 20 30 40 50 60
Clostridium tetani E88 (2)	M Q V N I N N L V S I S E A N Q N F S R V A R M V D E N G A A I I L K N N A P R Y V L I D Y S K F Q Q D T - - - - - I A D D A T V
Lactobacillus salivarius UCC118	M Q I N L E N L I P I T E A N Q N F S K V A R M V D S K G T A V I L K N N R P K Y V L V D Y N T L V Q E E Q T E A V V A D Q A S V
Streptococcus agalactiae CJB111	M Q I N I E N L V S I S E A N Q N F S K V A R M V D A N G T A V I L K N N T P K Y V L V D Y Q S L I K E E Q D T P V V V E Q T T L
Consensus	M Q i N i e N L v s I s E A N Q N F S k V A R M V D X n G t A v I L K N N X P k Y V L v D Y X X l X q e e q X X X v v a d q a t v

	70 80
Clostridium tetani E88 (2)	E E A A N N I L N K H L K A F E E L A K
Lactobacillus salivarius UCC118	D E V A S S I L S R H L E A L K E L A K
Streptococcus agalactiae CJB111	D E V A T S V L S R H L D A F K E L A K
Consensus	d E v A X s i L s r H L X A f k E L A K

7.

	10 20 30 40 50 60 70 80
Methanosarcina barkeri fusaro	M S T I A I D P D V K K S L K E F K I T E N E S Y N S I I K R L I V K V K E T A E Y K P M F P K E E N T E R R E S H V K D F D A W L D R K L I E D K E I L D A L G R K
Methanosarcina mazei Go1	M S T I A I D P D V K E S L K E L K L A P E E S Y N S V V K R L I G E V K K K E D Y S P M F P K E E K Q G H K E S H I N D F D A W L E K K L V E D K N I L D A L G R K
Consensus	M S T I A I D P D V K X S L K E X K X X X X E S Y N S X X K R L I X X V K X X X X Y X P M F P K E E X X X X X E S H X X D F D A W L X X K L X E D K X I L D A L G R K

8.

	10 20 30 40
Campylobacter jejuni TGH 9011	M K T T E Y N T Q Q K N N K E Y L A E R E A V K K N Y G K N G K R V R K T C Y K C G V S K T - - -
Campylobacter jejuni RM1221	M K T T E Y N T Q Q K N N K E Y L A E R E A V K K T M A K M E K E L E K P A I N A V F Q R L K N K
Consensus	M K T T E Y N T Q Q K N N K E Y L A E R E A V K K K K K

9.

```
                          10        20        30        40        50        60        70
Sulfitobacter sp. EE-36   MTRVTATEFKNNIGAFSDAAMSEPVIITSHQRDRLVLLSADEYRRLTALTEGVTAQDKDRVARGLERHRSTILELAKR
Sulfitobacter sp. NAS-14  MTRVTATEFKNNIGAFSDAAMSEPVIITSHQRDRLVLLSADEYRRLTALTEGVTAQDKDRVARGLERHRSTILELAKR

Consensus                 MTRVTATEFKNNIGAFSDAAMSEPVIITSHQRDRLVLLSADEYRRLTALTEGVTAQDKDRVARGLERHRSTILELAKR
```

10.

```
                              10        20        30        40        50        60        70        80
delta proteobacterium MLMS-1 (1)  MGKVLQVDNNFTPCPAQGGDELYPNGIFEFNITKILEEIQKKPASFSLAEAVVSDFPREFSSINESHLDSVDLSRPVILAEIAPG
delta proteobacterium MLMS-1 (2)  MGKVLQVDNNFTPCPAQGGDELYPNGIFEFNITKILEEIQKKPASFSLAEAVVSDFPREFSSINESHLDSVDLSRPVILAEIAPG

Consensus                          MGKVLQVDNNFTPCPAQGGDELYPNGIFEFNITKILEEIQKKPASFSLAEAVVSDFPREFSSINESHLDSVDLSRPVILAEIAPG

                                     90       100       110       120       130       140       150
delta proteobacterium MLMS-1 (1)  QYNLIDGHHRVEKARRLKIEKVPAYRLDVERHIKFLTSKRAYTAYIEYWNSKLTPGRKPRRKRGMAS
delta proteobacterium MLMS-1 (2)  QYNLIDGHHRVEKARRLKIEKVPAYRLDVERHIKFLTSKRAYTAYIEYWNSKLTPGRKPRRKRGMAS

Consensus                          QYNLIDGHHRVEKARRLKIEKVPAYRLDVERHIKFLTSKRAYTAYIEYWNSKLTPGRKPRRKRGMAS
```

11.

```
                                            10        20        30        40        50        60        70        80        90
Bacillus halodurans C-125 (1)           ------MPVPDRGNLVYVDFNPQSGHDQAGTRPAIVLSPKLFNKNTGFAVVCPITR-----QQKGYPFEIEIPPGLPIEGVILTDQVKSLDW
Escherichia coli K12                    ---MVSRYVPDMGDLIWVDFDPTKGSEQAGHRPAVVLSPFMYNNKTGMCLCVPCTT-----QSKGYPFEVVLS-GQERDGVALADQVKSIAW
Escherichia coli O157:H7                ---MVSRYVPDMGDLIWVDFDPTKGSEQAGHRPAVVLSPFMYNNKTGMCLCVPCTT-----QSKGYPFEVVLS-GQERDGVALADQVKSIAW
Leptospira interrogans lai 56601        -MVKNRNYTPEKGDIVWLNFTPQAGHEQKGRRPALVLSPKEYNSKTGLAIFCPITS-----KIKGYPFEVLIK-SKKIDGVILSDQVKNLDW
Moorella thermoacetica ATCC 39073 (1)   MVNEMETYIPERGDIVWLQFDPRAGHEQAGRRPALVISPQLYNGKVGLALFCPVTT-----KAKGYPFEVEIPAGLKITGVILADQVKSLDW
Neisseria gonorrhoeae FA 1090           ------MYIPERGDIFHLAFDPAAGTEMKGGHYAIALSPRAYNRATGLVYACPISQGRAAAARSGGMISTLLGTGTATQGNVHCHRMKALDW

Consensus                               ---XXXXyXPXXGdXXwXXFdPXXGXeqaGXrpAXvlSPXXyNXktGXXXXcPXtX-----XXkGypfevXXXXgXXXXGvXlXdqvKsldW

                                             100       110       120
Bacillus halodurans C-125 (1)           RARNFHIKGQAPEETVTDCLQLIHTFLS-
Escherichia coli K12                    RARGATKKGTVAPEELQLIKAKINVLIG-
Escherichia coli O157:H7                RARGATKKGTVAPEELQLIKAKINVLIG-
Leptospira interrogans lai 56601        TIREAEFIESINKVSLKEVLDNIKLLIF-
Moorella thermoacetica ATCC 39073 (1)   RAREAQFACKVPAGVVAEVQAKVQVLIS-
Neisseria gonorrhoeae FA 1090           KIRRAAFRETVPDYVIEDVLARIGAVLFD

Consensus                               raRXaXXXXXvXXXXXXXXXaXiXXliX-
```

12.

Sequence	10 · 20 · 30 · 40 · 50 · 60 · 70 · 80
Archaeoglobus fulgidus DSM 4304 (1)	-MKAVIDTNVIVYDTFEDSVFHQEAMQLLDR--IDVWVIPTIVIHEYVWVLKSLKVDVK-EIKYKVEEYLNHYKTKMVSENK
Pyrobaculum aerophilum IM2	-MKVLVDTNVLIYETFEDFERHAEATDIVY---TNEVYIPTIVLHEYIWLLLRHFSISYAQAAAKLEQLLSEKNIHVICENL
Pyrococcus abyssi GE5 (1)	-MHAVIDTDILIYDTFEDLEFHEEARALLDS--LSKWYVPTIVLQEYIWFFKRNNFSLL-DAKSMLMEYVRDPRFKGLGESH
Pyrococcus abyssi GE5 (2)	--MTVIDTNVFIYAILRDSEFNSRARNLLAS--LERWIVPSIVLYELYWFFREEGYGRD-EITNVISSILNSPRTRVIGDNG
Pyrococcus furiosus DSM 3638	-MHAVIDTNVLIYDTFSDSEFHKESRSLLNS--LDRWYIPSIVLQEYVWFFRSQGFSSR-EAKIMLSEYISDPRFRGLVEDH
Pyrococcus horikoshii OT3	------MHIVLIYDTFIDSEFHKKARSLLDS--LDRWYIPPTVLQEYVWFFRSQGFSSR-DAKIMLSEYIR----------
Sulfolobus acidocaldarius DSM 639	-MKILVDTNVLIYDLVENSKFHKEAEDILDR--AETWVIPSVVVHELVWFLRANNLER---MDYVSAYVESPKAEIVCEDF
Sulfolobus tokodaii 7 (2)	-----------MYSTFEDSERHKEAMKILT---ENEVVIPQIVVYEYIWVLARLTNN---VDLVKQKLEELKDFEIAKEDL
Sulfolobus tokodaii 7 (3)	MEKAIIDTNVIIYDYVEDSEYHKKAEELLDS--LNKWIIPAIVIHELVWFLKDMKLEDK---INDVFAYVRNEKAEVICDSV
Thermococcus kodakarensis KOD1 (1)	-MHAVIDTNVLLYDTFEDLPFHHKARRLLDS--LDRWYVPPIVLQEYVWFFRRKNLPVK-LARSMLSEYLDDPRFNRLNDNG
Thermococcus kodakarensis KOD1 (2)	-MEAVIDTNVFLYAAVEEMPRHREAFELLHSPSLEKWIVPTIVIYEVVWNFRKLGFSSE-EARELVEQIVEDERTKLVDDR-
Thermococcus kodakarensis KOD1 (3)	--MTVIDTNVFIYATLRDSEFNAEARKLLAG--LERWIVPSIVLYELYWFFREEGYKSE-EINNVISSILNSPRTRVICDTG
Consensus	-mXXvidtnvXiYdtfedsefhXeaXXllXs--lXXwXiPXiVlXEyvWfffrXXXXXXX-XXXXXXXXyXXXXXXXXXXXXX

Sequence	90 · 100 · 110 · 120 · 130 · 140
Archaeoglobus fulgidus DSM 4304 (1)	QIVLSALERIVGGGLSLSRYNDELILAVAGREKISLATFDERLRRQARARGVEVIP----
Pyrobaculum aerophilum IM2	SDLAAGIRMAAEDGAKPSNINDYIILASALNRGLALATYDRELRRAGARRAVTVLPATL-
Pyrococcus abyssi GE5 (1)	EVIIHALKILEENELSLSHFNDAIILYQAFSRKYPLATFDEKLRKLATKHGIRVLPEI--
Pyrococcus abyssi GE5 (2)	RYTKRALELTKNP-----RRFNDMIILATAEQFKRLATYDKRLKKEAERLGIETMP----
Pyrococcus furiosus DSM 3638	NVILRAIDILERENLSLSRFNDMIILVHAIE-KGTLATFDQKLRKLARKLSVEILP----
Pyrococcus horikoshii OT3	---------------------------------SKLTFSKGKTSLCHSLMI---------
Sulfolobus acidocaldarius DSM 639	DVLKNAMEIILREKISLSRYNDPVILSHAILRKLPLSTFDRRLSNLAKRYGIDIVK----
Sulfolobus tokodaii 7 (2)	EDMIKGIEMLKKDNKPIRMLNDYIILAIAKRLNIGLATYDIELVKAGVRNSVNIYSQHSS
Sulfolobus tokodaii 7 (3)	NNIVDSLEILIREKLPLADYKDMIILSHAIREKLPLVTFDKKLSKIAKKYGVSVVS----
Thermococcus kodakarensis KOD1 (1)	ESIVYALELIEKNSLSLSRFNDAIILYHALQRSYPLATFDKKFRKLAVKNGVEVLPAKV-
Thermococcus kodakarensis KOD1 (2)	RYLIKAFETLQSLSL---THYNDSVLLTIATEVGALATYDKKRRKRAKKLGIKLLPEVVE
Thermococcus kodakarensis KOD1 (3)	KYTKRALELTRNP-----KRFNDMVILATAEDFKRLATYDKRLKKEAEKLEIKTLP----
Consensus	XXXXXaXeXXXXXXXXXXXXndXXilXXaXXXXXXlaTfdXXlXkXaXXXgXXXXp----

13.

```
                                                 10        20        30        40        50        60        70        80
Photorhabdus luminescens laumondii TTO1  MIRVPSQYARSLDLRCCWSVFEHSAVSCRQISSSVEMKRMVGVKSCGFIDISVQVLNEVAHVCVRKLKMSWEEIAEFLDIV
Rhodobacter sphaeroides 2.4.1            ----------------MSAEFADTNVILYLLDN-GPKADCAEEILGHRPRISVQVLNETLVNCRRKAGLSWEEAGAFLEGV
Xanthomonas axonopodis citri 306 (2)     -------------------MFLDSNVVLYLLSEDAVKADGAEALLQRRPVISVQVLNEVTHVCVRKLKMGWDEVGQFLALV

Consensus                                ----------------XXXXFXdsnVXlyllsXXXXkadXaeXXlXXrpXISVQVLNEvXhvCvRKlkmsWeEXgXFLXXV

                                                90       100       110       120       130       140       150
Photorhabdus luminescens laumondii TTO1  RSFCKIVPLTVEVHDRARLIAERYRLSFYDSCIVAAAVIAGCRTLYSEDMNHGQMLEDSLVIKNPFSYV
Rhodobacter sphaeroides 2.4.1            RALCPVESLTIQTHDVGRALAERYGFSIYDSIIVASALVAGCTTLWSEDMQDGLLVEGQLRIVNPFA--
Xanthomonas axonopodis citri 306 (2)     RSFCKIVPLTVDVHDRARQLAERHQLSFYDACIVAAAAIEGCQTLYSEDMHHGLIIEESLSIRNPFNV-

Consensus                                RsfCkivpLTvXvHDraRXlAERyXlSfYDscIVAaAXiaGCXTLySEDMXhGlXXEXsLXIXNPFXX-
```

14.

```
                                   10        20        30        40        50        60        70        80
Sulfolobus tokodaii 7 (4)  -----------VDTSFLLPLVGIKVKGIKDSLLEGKAIYYPNLLLTELLAVIFKEAKKLKLNKVPEEAMKGLIYVLSNVN-LISIEELE
Sulfolobus tokodaii 7 (5)  MKRSKIVMRILIDTSFILPALGIDVG--EEIINIIKEFYNHEVYFTELSLLEAMWVIKRLIKQGIEVDFNVVKTGLKSINKTYRLVKIP

Consensus                  XXXXXXXXXXXXDTSFXLPXXGIXVXXXXXXXXXXKXXYXXXXXXTELXXXXXXXXXKXXXXXXXEXXXXXXXXXLXXXNXXXXXXXXX

                          90       100       110       120       130       140
Sulfolobus tokodaii 7 (4)  IETIYEILN-KGWNDIFDAILYTAYKSTKIPLITMDKSFYNFLKENGMDVKGIILL
Sulfolobus tokodaii 7 (5)  ISAYIKALNDKRHNDLIDLILYYTAKAYNLRLLSLDLKLKEIDKEN-IVIQSLNES

Consensus                  IXXXXXXLNXKXXNDXXDXILYXXXKXXXXXLXXXDXXXXXXXKENXXXXXXXXXX
```
