# Supplementary material for: TasA-tasB, a new putative toxin-antitoxin (TA) system from Bacillus thuringiensis pGI1 plasmid is a widely distributed composite mazE-doc TA system
Source: BMC Genomics. 2006 Oct 13;7:259. doi: 10.1186/1471-2164-7-259 (PMC1626090; doi:10.1186/1471-2164-7-259)
Supplement: Additional File 1 — Accession numbers. Accession number of all proteins (TasB homologues, TasA homologues, and their associated proteins) are listed in this table. [file 1471-2164-7-259-S1.pdf]

Strain	TasA	TasB
Alkaliphilus metalliredigenes QYMF	ZP_00799280	ZP_00799279
Anaeromyxobacter dehalogenans 2CP-C	YP_466077	YP_466078
Bacillus clausii KSM-K16 (1)	YP_174516	YP_174517
Bacillus thuringiensis H1.1 plasmid pGI1	not annotated	NP_705750
Bradyrhizobium sp. BTAi1	ZP_00863985	ZP_00863986
Brucella abortus 1 str. 9-941	YP_221320	YP_221319
Brucella melitensis 16M	NP_540292	NP_540293
Brucella suis 1330	NP_697574	NP_697573
Burkholderia thailandensis E264	not annotated	YP_442084
Candidatus kuenenia stuttgartiensis	CAJ74863	CAJ74864
Carboxydothermus hydrogenoformans Z-2901 (1)	YP_359137	YP_359138
Caulobacter crescentus CB15	not annotated	NP_422157
Chlorobium limicola DSM 245	ZP_00512710	ZP_00512679
Chlorobium phaeobacteroides BS1	ZP_00530611	ZP_00530390
Chlorobium phaeobacteroides DSM 266	ZP_00530064	ZP_00530036
Chlorobium tepidum TLS	NP_661236	NP_661237
Crocosphaera watsonii WH 8501	not annotated	ZP_00518660
Enterococcus faecalis V583	AAO80241	AAO80240
Enterococcus faecium DO (1)	ZP_00603841	ZP_00603842
Enterococcus faecium DO (2)	ZP_00605043	ZP_00605044
Geobacillus kaustophilus HTA426 (1)	YP_147698	YP_147699
Gloeobacter violaceus PCC 7421	NP_925680	NP_925681
Gluconobacter oxydans 621H plasmid pGOX2 (1)	not annotated	YP_190394
Halothermothrix orenii H 168	ZP_01189565	ZP_01189566
Jannaschia sp. CCS1	YP_511394	YP_511395
Lactobacillus acidophilus NCFM	YP_192967	YP_192968
Lactobacillus gasseri ATCC 33323 (1)	ZP_00046978	ZP_00046979
Mesorhizobium sp. BNC1	ZP_00614863	ZP_00614862
Nitrosomonas europaea ATCC 19718 (1)	not annotated	NP_842442
Nitrosomonas europaea ATCC 19718 (2)	not annotated	NP_841317
Nostoc punctiforme PCC 73102	not annotated	ZP_00111802
Oceanicola batensis HTCC2597	ZP_01000654	ZP_01000653
Parachlamydia sp. UWE25 (1)	YP_008021	YP_008020
Parachlamydia sp. UWE25 (2)	YP_008456	YP_008455
Pelodictyon phaeoclathratiforme BU-1	not annotated	ZP_00588413
Polaromonas naphthalenivorans CJ2	ZP_01020881	ZP_01020880
Prosthecochloris aestuarii DSM 271	ZP_00591716	ZP_00591629
Rhodobacter sphaeroides ATCC 17025 (1)	ZP_00912800	ZP_00912801
Rhodobacter sphaeroides ATCC 17025 (2)	ZP_00912210	ZP_00912211
Rhodoferax ferrireducens DSM 15236	YP_524645	YP_524644
Rhodopseudomonas palustris BisB18	YP_533817	YP_533816
Rhodopseudomonas palustris BisB5	YP_568550	YP_568551
Rhodopseudomonas palustris HaA2	YP_485053	YP_485054
Rhodospirillum rubrum ATCC 11170	YP_425070	YP_425071
Salinibacter ruber DSM 13855	YP_446906	YP_446907
Solibacter usitatus Ellin6076	ZP_00525003	ZP_00525004
Staphylococcus epidermidis RP62A	not annotated	YP_189114
Streptococcus pneumoniae TIGR4 (1)	AAK75015	AAK75016
Streptococcus pneumoniae TIGR4 (2)	ZP_00402572	ZP_00402571
Synechococcus sp. RS9917 (1)	not annotated	ZP_01081289
Syntrophomonas wolfei Goettingen (1)	not annotated	ZP_00664116
Xanthobacter autotrophicus Py2	ZP_01196919	ZP_01196918
Xanthomonas axonopodis citri 306 (1)	NP_641531	NP_641530
Xanthomonas campestris vesicatoria 85-10	YP_362953	YP_362952
Xanthomonas oryzae KACC10331	YP_202082	YP_202083
Xanthomonas oryzae MAFF 311018	YP_452272	YP_452273

Strain	TasA	Toxin
Archaeoglobus fulgidus DSM 4304 (1)	NP_071181	NP_071182
Archaeoglobus fulgidus DSM 4304 (2)	NP_070184	not found
Bacillus anthracis A2012	ZP_00390473	not found
Bacillus anthracis Ames	NP_842622	not found
Bacillus anthracis Ames Ancestor	YP_016656	not found
Bacillus anthracis Sterne	YP_026340	not found
Bacillus cereus ATCC 14579	AAP07157	not found
Bacillus cereus cytotoxis NVH 391-98	ZP_01180954	not found
Bacillus cereus G9241	ZP_00240570	not found
Bacillus clausii KSM-K16 (2)	YP_173562	not found
Bacillus clausii KSM-K16 (3)	YP_173588	not found
Bacillus halodurans C-125 (1)	BAB07439	BAB07440
Bacillus halodurans C-125 (2)	BAB03769	not found
Bacillus halodurans C-125 (3)	BAB03789	not found
Bacillus licheniformis ATCC 14580	YP_077342	not found
Bacillus sp. NRRL B-14911	ZP_01173381	not found
Bacillus subtilis subtilis 168	NP_387937	not found
Bacillus thuringiensis israelensis ATCC 35646	ZP_00739702	not found
Bacillus thuringiensis konkukian 97-27	YP_034407	not found
Bacillus weihenstephanensis KBAB4	ZP_01185739	not found
Carboxydothermus hydrogenoformans Z-2901 (2)	YP_359074	not found
Carboxydothermus hydrogenoformans Z-2901 (3)	YP_361415	not found
Clostridium acetobutylicum ATCC 824 (1)	AAK79903	AAK79902
Clostridium acetobutylicum ATCC 824 (2)	AAK81571	not found
Clostridium acetobutylicum ATCC 824 (3)	AAK81150	not found
Clostridium beijerincki NCIMB 8052	ZP_00909394	not found
Clostridium difficile QCD-32g58	ZP_01229320	not found
Clostridium perfringens 13	BAB82188	not found
Clostridium tetani E88 (1)	AAO34845	not found
Clostridium thermocellum ATCC 27405	ZP_00503889	not found
Desulfitobacterium hafniense DCB-2 (1)	ZP_00558166	not found
Desulfotomaculum reducens MI-1	ZP_01150217	not found
Escherichia coli K12	NP_417263	NP_417262
Escherichia coli O157:H7	NP_311670	NP_311669
Exiguobacterium sibiricum. 255-15	ZP_00540026	not found
Geobacillus kaustophilus HTA426 (2)	YP_145902	not found
Lactobacillus gasseri (2)	ZP_00046120	not annotated
Leptospira interrogans lai 56601	AAN48979	AAN48980
Moorella thermoacetica ATCC 39073 (1)	YP_429877	YP_429878
Moorella thermoacetica ATCC 39073 (2)	YP_428965	not found
Neisseria gonorrhoeae FA 1090	AAW89255	AAW89254
Oceanobacillus iheyensis HTE831 (1)	NP_690966	not found
Oceanobacillus iheyensis HTE831 (2)	NP_690985	not found
Photorhabdus luminescens laumondii TTO1	NP_928264	NP_928265
Pyrobaculum aerophilum IM2	AAL64466	AAL64467
Pyrococcus abyssi GE5 (1)	CAB49684	CAB49685
Pyrococcus abyssi GE5 (2)	CAB49041	CAB49040
Pyrococcus abyssi GE5 (3)	CAB50135	not annotated
Pyrococcus furiosus DSM 3638	AAL80697	AAL80698
Pyrococcus horikoshii OT3	NP_142833	NP_877801
Rhodobacter sphaeroides 2.4.1	YP_345380	YP_345381
Shewanella frigidimarina NCIMB 400	ZP_00640688	not found
Sulfolobus acidocaldarius DSM 639	YP_256551	YP_256550
Sulfolobus tokodaii 7 (2)	BAB65748	BAB65747
Sulfolobus tokodaii 7 (3)	BAB65851	BAB65852
Sulfolobus tokodaii 7 (4)	BAB67093	not annotated
Sulfolobus tokodaii 7 (5)	NP_377538	NP_377539
Symbiobacterium thermophilum IAM 14863 (2)	YP_077060	not found
Syntrophomonas wolfei Goettingen (2)	EAO24116	not found
Thermoanaerobacter ethanolicus ATCC 33223	ZP_00779727	not found
Thermoanaerobacter tengcongensis MB4 (1)	NP_621806	not found
Thermoanaerobacter tengcongensis MB4 (2)	NP_624083	not found
Thermococcus kodakarensis KOD1 (1)	BAD84646	BAD84645
Thermococcus kodakarensis KOD1 (2)	BAD86450	BAD86451
Thermococcus kodakarensis KOD1 (3)	BAD86418	BAD86417
Xanthomonas axonopodis citri 306 (2)	AAM37041	AAM37040

Strain	Antitoxin	TasB
Acinetobacter sp. ED45-25 plasmid pKLH205	not annotated	CAD31053
Aspergillus fumigatus Af293	not found	EAL85381
Azoarcus sp. EbN1	YP_159142 (aa 1 to 202)	YP_159142 (aa 203 to 324)
Bifidobacterium longum DJO10A	not found	ZP_00120313
Caldicellulosiruptor saccharolyticus DSM 8903	not annotated	ZP_00886106
Campylobacter jejuni TGH 9011	not annotated	AAS99045
Campylobacter jejuni RM1221	YP_179094	YP_179093
Chloroflexus aurantiacus J-10-fl	ZP_00766209	ZP_00766208
Clostridium tetani E88 (2)	not annotated	NP_782506
Corynebacterium diphtheriae NCTC 13129	not annotated	NP_939161
delta proteobacterium MLMS-1 (1)	ZP_01289550	ZP_01289540
delta proteobacterium MLMS-1 (2)	ZP_01290092	ZP_01290084
Desulfitobacterium hafniense DCB-2 (2)	not annotated	not annotated
Desulfitobacterium hafniense Y51	not annotated	YP_516864
Enterobacteria phage P1	YP_006570	YP_006571
Erwinia carotovora atroseptica SCRI1043	YP_048381	YP_048380
Ferroplasma acidarmanus Fer1	not found	ZP_00610578
Francisella tularensis novicida ATCC15482	AAP83346 (aa 1 to 182)	AAP83346 (aa 183 to 318)
Frankia sp. CcI3	YP_480200	YP_480201
Frankia sp. EAN1pec	ZP_00568300	ZP_00568301
Fusobacterium nucleatum vicentii	not found	ZP_00143594
Gluconobacter oxydans 621H (2)	not found	YP_192235
Haemophilus influenzae 86-028NP	YP_249090 (aa 1 to 218)	YP_249090 (aa 219 to 340)
Haemophilus influenzae R2866 (1)	ZP_00157327 (aa 1 to 218)	ZP_00157327 (aa 219 to 340)
Haemophilus influenzae R2866 (2)	ZP_00155060 (aa 1 to 210)	ZP_00155060 (aa 211 to 333)
Kineococcus radiotolerans SRS30216	not annotated	EAM76999
Lactobacillus reuteri 100-23	not annotated	ZP_01273906
Lactobacillus salivarius UCC118	YP_536276	YP_536275
Legionella pneumophila Lens	YP_125567 (aa 1 to 193)	YP_125567 (aa 194 to 319)
Magnetococcus sp. MC-1	not annotated	ZP_00606088
Mannheimia succiniciproducens MBEL55E	not found	YP_089359
Methanococcoides burtonii DSM 6242	not found	YP_564803
Methanosarcina barkeri fusaro	YP_303876	YP_303877
Methanosarcina mazei Go1	NP_632574	NP_632573
Mycobacterium avium paratuberculosis K-10	not annotated	NP_962078
Mycobacterium flavescens PYR-GCK	not annotated	not annotated
Mycobacterium sp. KMS	ZP_01282318	ZP_01282317
Mycobacterium sp. MCS	not annotated	YP_638009
Mycobacterium vanbaalenii PYR-1	not annotated	ZP_01207819
Mycoplasma mycoides SC PG1	not annotated	not annotated
Neisseria meningitidis MC58	AAF41326	AAF41325
Nocardioides sp. JS614	ZP_00659468	ZP_00659469
Nostoc sp. PCC 7120 plasmid pCC7120epsilon	NP_478675	NP_478676
Parachlamydia sp. UWE25 (3)	YP_008471	YP_008472
Pelobacter propionicus DSM 2379	not found	ZP_00678454
Pseudomonas aeruginosa PA01	not annotated	modified NP_248743
Psychrobacter sp. 273-4	YP_264166 (aa 1 to 198)	YP_264166 (aa 199 to 330)
Rhodopseudomonas palustris BisA53	not annotated	ZP_00809462
Rickettsiella grylli	not annotated	ZP_01300114
Salmonella enterica Choleraesuis SC-B67	YP_218475	YP_218474
Salmonella enterica Typhi Ty2	AAO69527	AAO69526
Salmonella typhimurium LT2	NP_462460	NP_462459
Shewanella baltica OS155	ZP_00583273	ZP_00583272
Shewanella oneidensis MR-1	NP_716019 (aa 1 to 189)	NP_716019 (aa 190 to 332)
Sodalis glossinidius 'morsitans'	not annotated	YP_455242
Streptococcus agalactiae CJB111	ZP_00787618	ZP_00787605
Sulfitobacter sp. EE-36	ZP_00956017	ZP_00956018
Sulfitobacter sp. NAS-14	ZP_00963618	ZP_00963619
Sulfolobus tokodaii 7 (1)	not annotated	BAB66181
Symbiobacterium thermophilum IAM 14863 (1)	not found	YP_074705
Synechococcus sp. RS9917 (2)	ZP_01081563	ZP_01081564
uncultured archaeon GZfos26G2	AAU83175	AAU83176
Vibrio cholerae O1 eltor N16961	not annotated	NP_232867
Vibrio cholerae RC385	ZP_00751723	ZP_00751724
Vibrio cholerae V52	not annotated	ZP_00744968
Vibrio metschnikovii CIP A267	AAN33027	AAN33028
Wolinella succinogenes DSM 1740	not found	NP_907321
Yersinia mollaretii ATCC 43969	ZP_00824096	ZP_00824095
Yersinia pestis CO92	NP_405398	NP_405399
Yersinia pestis KIM	not annotated	NP_669780
Yersinia pestis Medievalis 91001	AAS61794	AAS61793
Zymomonas mobilis ZM4	not annotated	modified NP_758996
